# Evidence of the Mechanism by Which Polyomaviruses Exploit the Extracellular Vesicle Delivery System during Infection

**DOI:** 10.3390/v12060585

**Published:** 2020-05-27

**Authors:** Simone Giannecchini

**Affiliations:** Department of Experimental and Clinical Medicine, University of Florence, I-50134 Florence, Italy; simone.giannecchini@unifi.it

**Keywords:** polyomaviruses, extracellular vesicles, microRNA, DNA viral load, viral persistence, polyomavirus-associated diseases

## Abstract

Increasing evidence suggests that human viruses can hijack extracellular vesicles (EVs) to deliver proteins, mRNAs, microRNAs (miRNAs) and whole viral particles during viral persistence in the host. Human polyomavirus (PyV) miRNAs, which downregulate large T-antigen expression and target host factors, help the virus escape immune elimination and may have roles in the success of viral persistence/replication and the development of diseases. In this context, several investigations have detected PyV miRNAs in EVs obtained from cell culture supernatants after viral infection, demonstrating the ability of these vesicles to deliver miRNAs to uninfected cells, potentially counteracting new viral infection. Additionally, PyV miRNAs have been identified in EVs derived from the biological fluids of clinical samples obtained from patients with or at risk of severe PyV-associated diseases and from asymptomatic control healthy subjects. Interestingly, PyV miRNAs were found to be circulating in blood, urine, cerebrospinal fluid, and saliva samples from patients despite their PyV DNA status. Recently, the association between EVs and PyV viral particles was reported, demonstrating the ability of PyV viral particles to enter the cell without natural receptor-mediated entry and evade antibody-mediated neutralization or to be neutralized at a step different from that of the neutralization of naked whole viral particles. All these data point toward a potential role of the association between PyVs with EVs in viral persistence, suggesting that further work to define the implication of this interaction in viral reactivation is warranted.

## 1. Introduction

Polyomaviruses (PyVs) are nonenveloped, small icosahedral viruses whose genomes consist of a single molecule of closed circular, supercoiled, double-stranded DNA. For decades, PyV JC (JCPyV) and PyV BK (BKPyV), the first two human isolates discovered in 1971, remained the only members of the family of PyVs in the human population [1,2]. Advances in DNA sequencing technologies have increased the number of PyVs identified to infect humans to fourteen [3,4]. Of note, among all PyVs identified in human population, simian virus 40 (SV40) and Merkel cell (MCPyV)-associated Merkel cell carcinoma are the other most studied PyVs [3,4]. PyVs, which share features such as their ability to establish long-term persistent infection via incompletely defined mechanisms, are a serious concern in immunocompromised human subjects. The main pathogenic mechanisms adopted by PyVs include (i) cytopathic pathology with high-level virus replication and without significant inflammation, as observed in progressive multifocal leukoencephalopathy (PML) caused by JCPyV; (ii) immune reconstitution inflammatory syndrome with a dominant inflammatory response to abundant PyV antigen in BKPyV-associated hemorrhagic cystitis; (iii) cytopathic inflammatory PyV pathology with PyV-associated nephropathy; (iv) autoimmune PyV pathology with a pathologic response to “self”, which may be triggered by viral antigens; (v) and oncogenic PyV pathology with PyV Merkel cell (MCPyV)-associated Merkel cell carcinoma [5].

Molecular investigations and serological studies have reported that JCPyV and BKPyV, but also the less extensively investigated novel PyVs, are ubiquitous and highly prevalent (up to 90% positivity) in the normal human population [6]. In this context, primary infections that are asymptomatic in healthy individuals and predominantly occur early in childhood with transmission mainly through an orofecal or respiratory route, are subclinical [6]. Human PyVs primarily persist in several tissues, including the kidney, bone marrow, and central nervous system (CNS), where they interact with cellular processes and modulate immune responses [7]. However, the mechanism adopted by these viruses to persist in the human population remains unclear.

Increasing evidence demonstrates that after cell infection, many viruses may hijack secretory extracellular vesicles (EVs) [8] to carry not only several viral components but also, in some instances, infectious viruses, providing additional routes of transmission and escape from immune recognition and facilitating viral persistence in the infected host [9,10,11,12]. In this context, investigating the molecular mechanism of the interaction between viruses and infected cells during the late phase of viral replication has been fundamental to elucidating the association of viruses with the EV secretory pathway to discern the role of this mechanism in viral persistence. Unfortunately, although many studies have investigated the mechanism of PyV entry, studies on the mechanisms of assembly and release of newly formed virions from infected cells are limited. In this article, the published literature on the features of the PyV–EV interaction are reviewed, with a focus on the potential role of this interaction during PyV infection on the ability of PyVs to persist in the host.

## 2. Polyomavirus Life Cycle and Extracellular Vesicles Biogenesis

The capsid of most PyVs, made of five copies of the major capsid protein Vp1 surrounding the minor proteins VP2 and VP3, is approximately 45 nm in size [13,14]. PyV capsids carry a double-stranded DNA genome of approximately 5 kb, which can be divided into (i) the early viral gene region (encoding the small (STAg) and large T-antigens (LTAg)), (ii) the late viral gene region (mainly encoding the capsid proteins Vp1, Vp2, and Vp3 and the agnoprotein), and (iii) the noncoding control region (NCCR) [3]. The NCCR harbors the origin of genome replication (*ori*) and promoter/enhancer regions with DNA-binding sites for transcription factors that mediate secondary host cell specificity [3] and control gene expression and viral DNA replication [5]. Variations in the NCCR are associated with virus reactivation and the high replication activity implicated in the development of PyV-associated diseases [3,4,5]. Additionally, several PyVs encode microRNAs (miRNAs; [15,16,17] nucleotide-long noncoding RNAs) with features similar to those of cellular miRNAs [18,19,20,21]. PyV miRNAs downregulate early viral gene expression and target host factors, aiding viral escape from immune elimination [22]. Finally, in the genomes of simian virus 40 (SV40) and BKPyV, histones have been found to be packed with the core (histones H2A, H2B, H3, and H4; [15,16]). 

**Figure 1:**
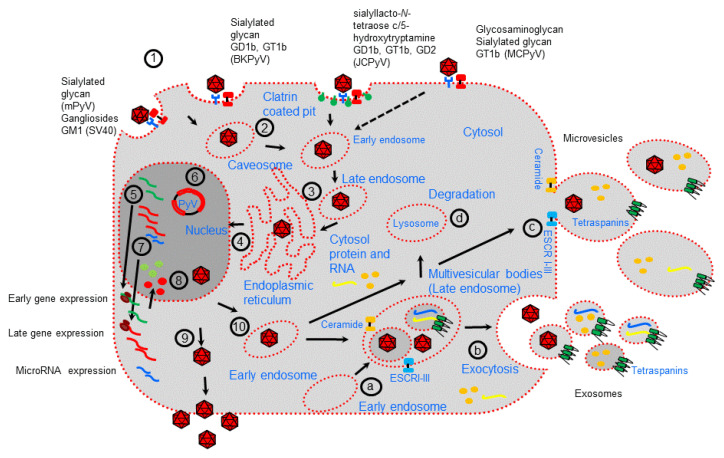
Polyomavirus life cycle and extracellular vesicle formation. Polyomavirus (PyV) infection starts with receptor-mediated interactions with specific receptors (**1**). Then, receptor-mediated endocytosis occurs mainly by internalization through caveosomes that traffic through the cytoplasm using a microtubule network to the late endosome before being delivered to the endoplasmic reticulum (ER) (**2**–**3**). JCPyV is internalized through clathrin-coated vesicles. In the ER, virions benefit from chaperones, disulfide isomerases, and reductases, which facilitate partial capsid uncoating (**3**). The viral genome is then transported into the nucleus via the nuclear pore complex (**4**). Expression of early genes occurs, and the proteins are translocated into the nucleus, where they serve to initiate viral DNA replication (**5**–**6**). Late genes are then expressed (**7**). Late proteins are translocated into the nucleus, where they self-assemble to form capsids into which newly synthetized viral DNA is packaged (**8**). During viral expression, PyVs also encode microRNAs involved in the regulation of early viral gene expression and target host factors. Progeny virions are mainly released from infected cells after cell lysis (**9**). However, a small fraction of progeny virions may also be released into the extracellular environment through nonlytic egress, which depends on the cellular secretion pathway generating extracellular vesicles (**10**). EVs are formed either (**a**–**b**) as early endosomes that accumulate intraluminal vesicles within the lumen of multivesicular bodies (MVBs) that fuse with the plasma membrane to release exosomes or (**c**) by budding of the plasma membrane (microvesicles). Early endosomes can fuse with lysosome to fulfill the degradation pathway (**d**).

The PyV life cycle starts with attachment of the Vp1 capsid protein to target cells mediated by cellular receptors identified by the common use of gangliosides as primary receptors for many PyVs (Figure 1; [17,23]). Thus, although it has been suggested that different PyV bind a distinct spectrum of cell surface receptors having different cellular tropisms, several studies have reported that gangliosides, one type of glycosphingolipid that are enriched within the lipid raft portions of cell membrane, play an important role in the initial interaction between PyV and cell substrate. Additionally, pentasaccharide lactoseries tetrasaccharide c, for which the serotonin receptor 5HT2A acts as a possible coreceptor, was also observed to act as a receptor for JCPyV [24]. MCPyV binds GT1b and uses glycosaminoglycans as possible coreceptors [25].

Except for JCPyV, which is internalized by clathrin-mediated endocytosis, internalization of PyVs is predominantly mediated by caveolar/lipid raft-mediated endocytosis [26]. PyV particle uncoating starts after PyV particle transfer into the endoplasmic reticulum (ER), followed by translocation across the ER membrane and uses the ER-associated degradation machinery [26,27]. In this action, it is also possible there is viroporin activity of Vp2/Vp3 [26,27]. In this mechanism of virus internalization, cellular interaction factors such as the tetraspanin CD63, syntenin-1, and the endosomal sorting complex required for transport (ESCRT) protein ALIX may be involved, as described for other viruses [28].

After PyV DNA enters the nucleus via an incompletely understood mechanism [29], transcription and replication of the viral genome occur, governed by the NCCR. In particular, transcription of the early viral gene region is initiated prior to the onset of viral DNA replication, followed by transcription of the late viral gene region [30]. The early and late viral gene regions are transcribed in opposite directions, which is also mediated by cellular host factors. Early transcription generates a single precursor mRNA from which different transcripts are generated through alternative splicing, generating the regulatory proteins LTAg and STAg [30]. The late region encodes at least two capsid proteins, VP1 and VP2, which are translated from differently spliced mRNAs [31]. The genomes of most PyVs (except MCPyV) contain an open reading frame (ORF) for a third structural protein, VP3, which can be translated from the same mRNA used to translate the VP2 protein by the use of an internal in-frame start codon. Of the known human PyVs, SV40, BKPyV, and JCPyV encode additional Vp4 proteins. In SV40, BKPyV, and JCPyV, a gene encoding agnoprotein resides in the late region immediately upstream of the VP1 gene [32,33,34]. Agnoprotein is involved in viral transcription, DNA replication, and virion biogenesis and release [33]. Viral DNA replication requires the presence of the viral protein LTAg which, as a double hexamer, binds and recruits cellular proteins required for DNA replication [35]. The additional viral proteins STAg and agnoprotein have auxiliary functions [36,37].

Expression of PyV miRNAs starts in the nucleus with the transcription of a primary miRNA (pri-miRNA), which is recognized and cleaved by the Drosha–DGCR8 microprocessor complex into a precursor miRNA (pre-miRNA, approximately 60 nt long) that, after export into the cytoplasm by the protein exportin 5, is further processed into a 22 nt double-stranded miRNA duplex (made of the miRNA-5p and miRNA-3p strands) [20,38]. One strand of this miRNA duplex (mainly the miRNA-5p guide strand) is incorporated into the RNA-induced silencing complex (RISC), comprising Dicer, TRBP (a dsRNA-binding domain protein), and Argonaute protein 2 (Ago2), and finally becomes a mature miRNA [20,38].

Although viral particles are assembled in the nucleus [39], the mechanism underlying viral particle release is not completely understood. PyVs can be released from infected cells, which may or may not cause cell lysis [40,41]. In this context, the agnoprotein seems to play a role in virion assembly and release [42,43,44,45]. Notably, whereas the JCPyV agnoprotein possesses viroporin activity [46], the BKPyV agnoprotein can interact with α-soluble N-ethylmaleimide-sensitive fusion attachment protein (α-SNAP), suggesting an interference mechanism with the exocytotic pathway [47].

Examination of the PyV life cycle reveals molecular factors that regulate endocytosis and the exocytosis pathway that are common to the early and late stages of cellular viral infection [48,49,50]. Notably, as well described, the cellular exocytosis pathway is implicated in EV generation. EVs are a heterogeneous group of membrane vesicles secreted by almost all cell types. EVs are thought to have an important role in discarding unwanted material (proteins, nucleic acid), signaling vehicles in normal cell homeostatic processes, as T-cell stimulation and immune tolerance or may be produced as a consequence of pathological developments [51,52,53,54,55]. EVs can be categorized into multiple classes based on the main mechanism of their generation [51]. Among the different types of EVs are exosomes (vesicles 30–150 nm in size), which are produced by the endocytic pathway, accumulate into large multivesicular bodies (MVBs), and are mainly delivered by fusion with the cell membrane; ectosomes or microvesicles (vesicles 100–1000 nm in size), which are formed by direct budding from the plasma membrane or released by the fusion of double-membraned autophagosomes with the plasma membrane; and apoptotic bodies (vesicles 100–5000 nm in size), which are released upon cell fragmentation during apoptotic cell death [52,53,54]. MVBs produced by early endosomes accumulate intraluminal vesicles (exosomes), which can fuse with lysosomes for use in the degradation pathway or fuse with the cellular membrane, releasing exosomes into extracellular space (Figure 1; [54]). Distinct processes, high enrichment in tetraspanin molecules as well as endosome membrane reorganization and ESCRT recruitment to the site of exosome formation, are associated with the formation of exosomes within MVBs [55,56,57,58]. ESCRT-0 clusters ubiquitinated proteins for delivery into MVBs and recruits ESCRT-1 to the endosomal membrane, which subsequently enrolls ESCRT-II (which mediates membrane invagination and exosome formation) and ESCRT-III [59,60,61,62,63]. However, MVB biogenesis and exosome release using ESCRT-independent mechanisms have been reported, such as the syndecan–syntenin–ALIX pathway or through the inward budding of the limiting membrane of the MVBs which requires sphingolipid ceramide (a lipid-mediated mechanism) [64,65]. Alternatively, the ESCRT-independent mechanism of exosome biogenesis was shown to require ceramide, which allowed the generation of membrane subdomains for spontaneous negative cur-vature of the membranes [66]. Finally, once MVBs are formed, their fusion with the plasma membrane is mediated by the cytoskeleton, fusion machinery (such as the SNARE proteins), and molecular switches (such as small molecular weight GTPases), or MVBs may be released through budding from the plasma membrane independent of Rab GTPases [67,68].

Microvesicle biogenesis requires several molecular rearrangements mediated by lipid components (mainly cholesterol) and proteins of the plasma membrane [69]. In particular, Ca^2+^-dependent enzymes, including aminophospholipid translocases, scramblases, and calpain, mediate the exposure of phosphatidylserine from the inner leaflet to the cell surface, restructuring of the underlying actin cytoskeleton, and the formation of microvesicles [70,71]. Moreover, the activities of the Rho family of small GTPases and Rho-associated protein kinase, which are important regulators of actin dynamics, are also involved [72]. Additionally, the use of ceramide-dependent mechanisms as well as ESCRT I-III molecules has been reported in microvesicle formation, showing the common role of microvesicle formation and exosome generation in EV biogenesis.

EVs can carry various cargoes, including proteins, lipids, and nucleic acids, directly affecting their role and function ([51,52,53,54,73,74,75]; Table 1). According to the exosome content database ExoCarta, several proteins, lipids, mRNAs, and miRNAs have been identified in exosomes obtained from different cell types [75]. In this context, it was recently reported that miRNAs are incorporated into exosomes through four potential selective packaging mechanisms [76]: 1—the neural sphingomyelinase 2 (nSMase2)-dependent pathway [77]; 2—the miRNA exomotif and SUMOylated hnRNP-dependent pathway [78], in which SUMOylated hnRNP family proteins recognize specific nucleotide motifs of the miRNA sequence and guide its specific packaging into exosomes; 3—the miRNA sequence-dependent pathway [79], in which the miRNAs preferentially sorted into exosomes have more poly(U) bases than poly(A) bases at their end; and 4—the miRISC-related pathway [80].

miRISCs colocalize with the sites of exosome biogenesis (MVBs) and their components, such as the Ago2 protein and miRNA-targeted mRNA, and are correlated with the sorting of miRNAs into exosomes. Although the mechanism remains unclear, nucleic acids in microvesicles are targeted to the cell surface, likely involving conserved zipcode RNA sequence motifs in the 3ʹ untranslated regions of mRNAs [81]. Additionally, short amino acid motifs have been implicated in ESCRT-dependent protein recruitment into vesicle membranes [82,83]. The three classes of this motif, called “Late domain”, also implicated in viral proteins involved in ESCRT recruitment are all proline-rich motifs (PRMs) with the form PPXY, P(S/T]AP, or YPX_1/3_L [82,83]. The association of cytosolic components with microvesicles requires their binding to the inner leaflet of the plasma mem-brane mediated by plasma membrane anchors (palmitoylation, prenylation, myristoylation) [84,85].

## 3. Polyomavirus Association with Extracellular Vesicles

In recent years, an increasing number of investigations have reported that several PyVs encode miRNAs that silence gene expression by transcript-specific target-mediated inhibitory activity and play a key role in several cellular processes [18,19,20,21,22]. These PyV miRNAs downregulate early viral gene expression and target host factors aiding escape from immune elimination [22]. The principal target of PyV miRNAs is the large T-antigen mRNA, which results in the downregulation of viral replication [86,87,88,89,90]. Moreover, host factors identified as additional targets of PyV miRNAs mainly involved in the viral escape detection by the innate and adaptive immune systems include (i) the mRNAs of the stress-induced ligands ULBP3 and ULBP1, implicated in the NKG2D-mediated killing of virus-infected cells by NK cells [91,92]; (ii) the mRNA of the proapoptotic factor Smad2 and the protein AMBRA1, involved in the suppression of apoptosis and in autophagy [93,94]; (iii) the mRNA of the dual-specificity protein phosphatase DUSP8, the mRNA of the transcription factor RUNX1, the splicing factor RBM9/FOX2, and the repressor MECP2 [95,96]. Because several viruses use small EVs to deliver viral miRNAs, the potential use of EVs by PyVs has been investigated (Table 2). With the growing interest in investigating EVs and PyVs, a minimal technique required for EV purification and characterization has been of central importance [97,98,99]. Several methodologies have been used to isolate and analyze EVs from complex biological fluids [98]. However, the isolation and subsequent characterization of EVs remains difficult. To this end, numerous protocols and commercially available reagents have been used to purify EV from heterogeneous biological samples, including differential ultracentrifugation and several commercially available systems [99]. At the same time, characterization of EV-obtained particles has been performed using several methods, including nanoparticle tracking analysis (NTA), Western blotting (WB), and immunoelectron microscopy [99]. Although not all PyV and EV studies have used the same methods, rendering the data difficult to compare, collectively, the data confirmed the positive association between EVs and PyVs. Additionally, among the EV markers reported in Table 1, studies on PyVs have shown the minimal markers used to identify EVs. The in vitro expression and association the JCPyV miRNAs JC-miRNA-J1-3p and JC-miRNA-J1-5p with EVs at early time points postinfection were investigated in hematopoietic progenitor KG-1 cells and kidney fibroblast-like COS-7 cells transformed with SV40 after infection with a JCPyV archetype viral clone (Table 2; [100]). JCPyV miRNAs were investigated in characterized small EVs (identified as exosomes) obtained from cell-free supernatants at 24 h of JCPyV cell infection. NTA of small EVs obtained from the supernatants of KG-1 and COS-7 cells using an exosome-like purification kit showed a main particle size of 112 nm and a mean total particle concentration of 10^7^/mL. Moreover, EVs were positive for the specific exosome protein marker tetraspanin CD63, as revealed by WB [100]. The EVs obtained from both cellular systems showed JCPyV miRNA expression starting at 12 h postinfection but exhibited different kinetics of their expression at later times compared to that observed in cells [100]. Then, a study verified that JCPyV miRNAs in small EVs in the supernatants of infected cells might be carried into uninfected cells [100]. This data confirmed for PyV the previous evidence reported for Epstein–Barr virus that EV delivery of viral miRNA may act in transferring gene silencing activity from infected cells to a recipient cell [101]. Similar results were obtained in another study of BKPyV using the same technology to extract and characterize EVs from biological fluid (EVs with a main size of 112 nm, total particle concentration of 10^7^/mL, and positivity to CD63, CD81, and annexin II; Table 2; [101]). In this viral setting, the viral miRNAs bkv-miR1-B1-3p and bkv-miR1-B1-5p were expressed in small EVs derived from the supernatants of COS-7 cells and renal proximal tubule epithelial cells (RPTECs), the natural target of BKPyV infection in the human host. Moreover, in this study, the expression of viral miRNA was dependent on the nature of the viral NCCR. In particular, lower BKPyV miRNA expression was observed in the context of a rearranged NCCR critically linked to inactivation of a single Sp1-binding site (*sp1-4*) in comparison to their expression with the archetypal NCCR [101]. Finally, BKPyV-miRNA-5p enclosed in small vesicles obtained from BKPyV-miRNA-5p-expressing vector-transfected cells was demonstrated to be delivered to uninfected cells to counteract new BKPyV infection [102]. In the context of a clinical setting, although several studies reported the positive detection of PyV miRNAs circulating in biological fluid, few studies have specifically conducted an investigation of PyV miRNA in EVs [38]. Among these, some focused on the presence of viral miRNA in small EVs in biological fluid. Thus, JCPyV miRNAs were found to be associated with EVs obtained from the plasma and cerebrospinal fluid (CSF) of HIV-infected patients (Table 2; [103]) and plasma and urine from multiple sclerosis patients in the presence or absence of natalizumab therapy and healthy subjects [104]. In HIV patients, JCPyV miRNA in EVs was found in 6% of plasma samples in the absence of PML, with a positivity of 43% in serum samples and of 60% in CSF samples from PML patients [103]; in multiple sclerosis patients in the presence or absence of natalizumab treatment, EVs containing JCPyV JC-miR-J1-3p and JC-miR-J1-5p were found in 25–58% and 17–50% of plasma samples, respectively. Moreover, JCPyV JC-miR-J1-3p and JC-miR-J1-5p in EVs derived from the urine of multiple sclerosis patients in the presence or absence of natalizumab treatment exhibited a positivity rate of 26–58% and 19–25%, respectively (Table 2; [104]. Finally, 19–25% of plasma and urine samples from healthy controls exhibited JCPyV JC-miR-J1-3p and JC-miR-J1-5p positivity. In all, the association of PyV with EVs was found in not only viral DNA-positive samples but also viral DNA-negative samples. In another study, BKPyV miRNA expression associated with EVs in urine samples was investigated in multiple sclerosis patients (Table 2; [102]). Among these patients, although absent BKPyV-associated diseases, those that shed BKPyV with rearranged NCCRs had lower miRNA-5p levels in EVs than patients that shed BKPyV with the archetypal NCCR architecture, who showed higher bkv-miR-B1-5p levels in urinary small EV preparations [102]. Moreover, it was reported that BKPyV miRNA was highly abundant in urinary EVs in patients with BK virus nephropathy (Table 2; [105]. However, although direct correlation between EV PyV miRNA and PyV pathology was suggested in the latter study, the indirect correlation between PyV miRNA and viral load in the other study highlights the potential clinical relevance of PyV miRNAs in the risk assessment of PyV diseases [102,103,104]. Additional studies using the same technology, i.e., methods involving the extraction of EVs from biological fluid, have reported the presence of the PyVs BKyV, JCPyV, MCPyV, and SV40 in samples obtained from EVs derived from the saliva samples of HIV patients and healthy subjects (Table 2; [106]. In particular, PyV miRNA in EVs was found in 15–61% and 14–70% of saliva samples from HIV patients and healthy subjects, respectively, and both groups of subjects showed high levels of BKPyV miRNA [106]. In this context, the prevalence of PyV miRNA in saliva was higher than that in paired plasma samples [106]. Thus, it was suggested that PyV miRNAs delivered in saliva can be a marker of PyV persistence in the oral cavity [106].

With increasing evidence of the association of PyV miRNA expression in EVs delivered in biological fluid, the finding that EVs can deliver whole PyV particles also emerged (Table 2, [107,108,109,110]. However, it is important to note that the extremely complicated procedure used to separate EV preparation from viral particle could represent a limitation for such studies. This phenomenon was first documented for JCPyV [107]. EVs were extracted by ultracentrifugation, and NTA and WB were used to identify the molecular markers (CD9, CD81, annexin V, flotillin-1, TSG101) typical of these vesicles [106]. Moreover, immunomicroscopy showed the presence of viral particles inside the EVs but also outside and adsorbed to the extracellular membrane of EVs [107]. In this study, the association of JCPyV and EV during SVG-A cell infection was reported. A JCPyV pathogenic variant carried by EVs was demonstrated to evade antibody-mediated neutralization, suggesting its role in dissemination within the CNS [107,108]. Moreover, using mutant pseudoviruses defective in sialic acid receptor binding, purified pseudovirions were shown to be able to transduce cells only if associated with EVs. This may be important for the transmission of JCPyV into uninfected cells by a new mechanism, which occurs through the attachment of EV-associated virus to the host cells without interacting with natural virus cell receptors [112,113,114]. Moreover, the association of JCPyV with EVs in primary human choroid plexus epithelial (CPE) cells was recently reported (Table 2, [108]. In this study, EVs produced by CPE cells infected with JCPyV possessed characteristics and expressed EV markers (CD9, CD81, annexin V, flotillin-1, TSG101) similar to those of uninfected cells. Transmission electron microscopy of EVs from infected CPE cells showed viral particles bound to the outside of EVs and enclosed within EVs. These EVs were able to transmit the infection to human glial cells independent of the virus attachment receptor toward the inside using both clathrin-dependent endocytosis and macropinocytosis [108]. Additionally, as demonstrated in a previous study, infection with EVs was not neutralized by antisera directed against the viral Vp1 capsid protein [106,108]. The association of PyVs with EVs has also been reported for BKPyV (Table 2, [109]). In this study, EVs were obtained from supernatants of Vero- and RPTE-infected cells using a gradient ultracentrifugation protocol. Analysis of the molecular markers (CD9, CD63, CD81) and electron microscopy confirmed the nature of EVs and their association with BKPyV [109]. In particular, electron microscopy revealed the presence of multiple BKPyV particles (up to 10 viral particles) inside the EVs. Unlike the naked virions, the EV-associated particles did not use cell surface receptors and different entry pathways for infectious particles. Moreover, like the naked BKPyV virion, the EV-associated BKPyV particles were sensitive to antibody neutralization, but this occurred in the postattachment step after endocytosis [110]. Finally, in the context of a clinical setting, recently JCPyV has been investigated in EVs derived from HIV-positive patients and healthy controls (Table 2, [111]. The EVs were extracted by using an exosome-like purification kit, and NTA showed a particle size of up to 150 nm (with a peak at a diameter of 60–75 nm) and a mean total particle concentration of 10^9^/mL. Moreover, WB analysis and immunomicroscopy reported that the EVs were highly enriched in the tetraspanins CD63 and CD81 and in annexin II. Immunoelectron microscopic imaging also confirmed the presence of Vp1 proteins, at least in some EVs. Examination of plasma-derived EVs reported the detection of JCPyV DNA in 15 out of 36 (42%) of JCPyV DNA plasma viremic samples (with only 1 from a healthy control) at a mean level of 23.5 copies/mL. In total, EV-selected samples corresponded to 5.4% of the total JCPyV DNA viral load [111]. Noteworthy, in this study, EV treatment with DNase had no effect on the amount of JCPyV DNA measured in the purified EVs, thus excluding the possibility that external naked DNA linked to vesicles was co-purified with EVs during the purification step. Additionally, plasma treatment with Triton X-100 before EV purification verified the absence of detected JCPyV-DNA, excluding the possibility that JCPyV particles were co-purified with EVs during the purification step [111].

Although different EVs have been associated with different viruses, the PPXY and YPX_1/3_L amino acid motifs are the main classes of late assembly domains present in viral proteins that, by interacting with host factors, are involved in the endosomal sorting complexes responsible for the transport pathway in the shedding of EVs from the plasma membrane for different types of viruses [82,83]. Notably, the role of late assembly domains in the major capsid protein of the hepatitis A virus genome has been reported to explain the molecular mechanism of virus association with EVs [115]. Although the “late domains” have been implicated in ESCRT-dependent protein virus recruitment into vesicle membranes [82], the mechanism that permits PyV association with EVs is not understood. The fact that the PyV Vp1 major capsid protein possesses conserved late domain YPX_3_L amino acid motif (positions 300–305, BKPyV amino acid sequence numbering) among different homologous PyV Vp1 sequences [6] highlights the potential role of this protein in viral association with EVs and suggests that its role requires investigation.

## 4. Concluding Remarks

**Figure 2:**
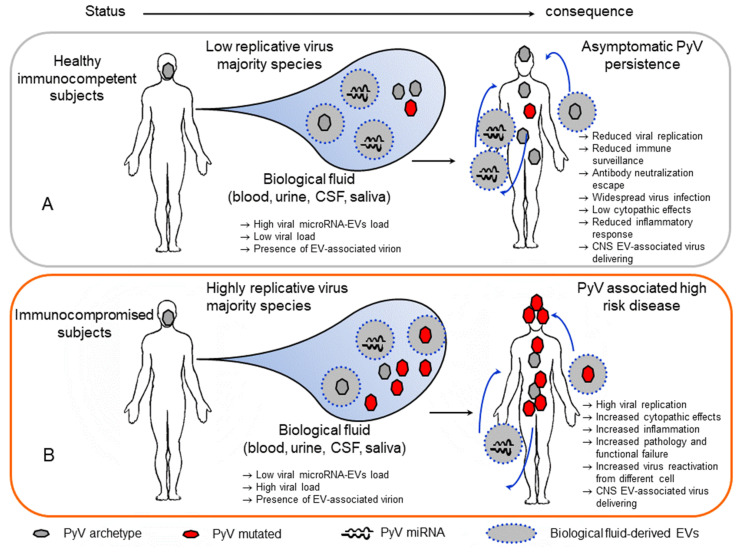
Potential role of extracellular vesicles in polyomavirus infection. (**A**) In healthy immunocompetent subjects, polyomaviruses persist in different tissues. In these subjects, replication is controlled by immune surveillance, microRNA autoregulation activity on large T-antigen and immune molecules within the host cell. Additionally, extracellular vessels carrying the viral microRNAs produced in infected cells can deliver these microRNAs to a noninfected or infected recipient cell, counteracting viral replication and increasing the downregulation of immune surveillance. Additionally, a few polyomavirus particles associated with extracellular vesicles may be present in the biological fluid and escape the neutralization mechanism, reduce cell cytotoxicity, and cause widespread infection. In this way, EV-associated viruses can be delivered into the CNS. (**B**) In immunocompromised subjects, reduced immune surveillance induces high viral replication. In this context, extracellular vesicles with produced microRNAs can partially counteract virus replication. Additionally, highly replicative viruses can be delivered into the CNS.

To date, increasing evidence suggests that the use of EVs as an intercellular communication mechanism may be exploited by several viruses during their persistence in the host [9,10,11,12]. EV-mediated transfer of genomes, mRNAs, miRNAs, proteins, or whole viral particles has been reported for different viruses [116]. In this context, the delivery of EVs carrying viral miRNAs or proteins to uninfected cells during virus infection plays a role in modulating the host immune response [12,116]. Additionally, the association of many virus particles with EVs may provide additional routes of transmission and escape from immune recognition and facilitate their persistence in the infected host [116]. EVs are thus speculated to play a role in PyV persistence in the host (Figure 2). 

Thus, it is possible that in healthy immunocompetent subjects, PyVs carrying the archetypal NCCR are transmitted and establish asymptomatic persistence at a different site of the host under the control of immune surveillance. In these subjects, viruses exhibit reduced replicative activity due to viral miRNA autoregulatory mechanisms and their downregulation of the immune response within infected cells. Additionally, EV-associated PyV miRNAs delivered from infected cells into uninfected cells or infected cells by an *in trans* mechanism may increase viral autoregulation and, at the same time, increase the downregulation of the immune response inducing the viral escape detection by the innate and adaptive immune systems. The effect may be of major importance for the minority of cells infected with PyV, reducing their ability to replicate and expand among the viral variant population in the host. Additionally, the presence of whole-JCPyV particles into EVs potentially generated during persistence in the kidney or bone marrow, albeit at a reduced percentage, could be implicated in the transport of virus in the circulation with the potential to deliver virus to the CNS and evade antibody neutralization. Notably, this strategy has been reported to be used by other viruses (i.e., HEV, HAV, and picornavirus) to persist in the host [115,117,118,119,120]. The delivery of PyVs in EVs also supports the possibility to infect different susceptible and unsusceptible cells, increasing the positive cellular distribution of the virus. Additionally, as reported for hepatitis E virus HEV, the transport of PyVs in EVs could be a strategy used by the virus to reduce the level of danger signals produced by cell lysis during virus egress from infected cells and to modulate the inflammatory response [120]. This latter mechanism could be relevant for PyV enabling low level dissemination of infectious virus to uninfected cells to establish lifelong persistent infections in their natural hosts [41]. Notably, as reported for poliovirus, coxsackievirus, and rhinovirus, the delivery of multiple virus types carried by EVs demonstrated that infection with PyV in EVs allows for a significantly greater replication efficiency than infection with a similar number of viral particles not embedded in a vesicle [108,109,118]. Conversely, in immunocompromised subjects in whom the virus can increase its replicative activity, EVs carrying viral miRNAs from archetype variant-infected cells are no longer able to control the high replication rate of the mutated PyV form (Figure 2B). At the same time, virus reactivation from different cellular sites previously not susceptible to virus replication may increase viral spread and the development of PyV-associated diseases. Additionally, as hypothesized in a recent study, JCPyV associated with EVs produced by CPE cells may be a main mechanism of JCPyV delivery to the brain due to the role of CPE cells in blood–brain communication and cause PML [108].

Several intensive efforts have elucidated some aspects of the biological pathway adopted by PyVs to persist in the host, but the mechanisms used by PyVs to spread to such a wide range of organs and/or tissues in the infected host have not been determined [121]. New investigations of the association of PyVs with circulating EVs in humans are warranted to shed new light on their role in PyV-associated disease and their immunoregulatory potential and to develop new antiviral strategies.

## Figures and Tables

**Table 1:** Main characteristics of extracellular vesicles.

Features and Markers	Extracellular Vesicles	
	Exosomes	Microvesicles
Origin	endosome	Plasma membrane
Size	30–200 nm	100–1000 nm
Membrane markers	Tetraspanins: CD9, CD81 CD63, TSPAN6, TSPAN8, CD151, CD37, CD53, Flotilin 1 and 2	Tetraspanins: CD9, CD63, CD81, CD82
Lipids	Phosphatidylserine, cholesterol, ceramide and other sphingolipids, LBPA	Phosphatidylserine, ceramide phosphatidylethanolamine, sphingolipids
Cell adhesion	Integrin, lactadherin, ICAM	Integrin, PECAM1, fibronectin
Intracellular trafficking	Rab GTPases, annexins	Rab GTPases, annexins
Cell type-specific protein	MHC-I, MHC-II, APP, PMEL, TCR, FasL, CXCR4, HSPG, CD86, PrP, TFR, WNT	MHC-I, MHC-II, APP, PMEL, TCR, FasL, CXCR4, HSPG, CD86, PrP, TFR, WNT LFA1, CD14
Cytoplasmic material (enzyme)	Peroxidases, pyruvate kinase, enolase, GAPDH	Tau, TDP43, GAPDH
Signaling molecules	Protein kinases, catenin, 14-3-3, G proteins	For example, ARF6, RAB11, ROCK
Biogenesis components	ALIX, TSG101, syntenin, ubiquitin, clathrin, VPS32, VPS4	ALIX, TSG101, ERK, PLD, VPS4
Chaperones	HSP70, HSP90	HSP70, HSP90
Cytoskeletal molecules	Not determined	Actin, tubulin
Nucleic acids	MicroRNAs and other noncoding RNAs, mRNA, DNA (and histones) (associated to the outside of the EV or part of their cargo)	MicroRNAs and other noncoding RNAs, mRNA, DNA (and histones) (associated to the outside of the EV or part of their cargo)

ALIX, ALG-2-interacting protein X; APP, amyloid precursor protein; ARF6, ADP-ribosylation factor 6; ARMMs, arrestin-domain-containing protein 1-mediated microvesicles; CXCR4, chemokine receptor 4; GAPDH, glyceraldehyde-3-phosphate dehydrogenase; HSP70, heat shock 70 kDa protein; HSPG, heparan sulfate proteoglycan; ICAM, intercellular adhesion molecule; LBPA, lyso-bis-phosphatidyl acid; LFA1, lymphocyte function-associated antigen 1; MHC, major histocompatibility complex; PECAM1, platelet endothelial cell adhesion molecule; PLD, phospholipase D; PrP, prion protein; ROCK, Rho-associated protein kinase; TCR, T-cell receptor; TDP43, TAR DNA-binding protein 43; TFR, transferrin receptor; TSG101, tumor susceptibility gene 101 protein; TSPAN, tetraspanin; VPS, vacuolar protein sorting-associated protein [69,70,71,72].

**Table 2:** Polyomavirus and extracellular vesicles (EVs) reported in studies.

Polyomavirus	Biological Fluid	EV Extraction and Characterization Methods	EV Markers	PyV Markers	Reference
Studies on EVs and MicroRNAs					
*JCPyV*	COS-7 cell supernatant, KG-1 cell supernatant	Exosomes extraction kit; NTA and WB	CD63	jcv-miR-J1-3p and -5p	[100]
*BKPyV*	COS-7 cell supernatant, RPTEC supernatant	Exosomes extraction kit; NTA and WB	CD63, CD81, annexin II	bkv-miR-B1-3p and -5p	[102]
*JCPyV*	Plasma Urine Saliva CSF	Exosomes extraction kit; NTA and WB	CD63,	jcv-miR-J1-3p and -5p	[103,104,105,106]
*BKPyV*	Plasma Urine Saliva	Exosomes extraction kit		bkv-miR-B1-3p and -5p	[105,106]
*MCPyV*	Plasma Saliva	Exosomes extraction kit		mcv-miR-M1-5p	[106]
*SV40*	Plasma Saliva	Exosomes extraction kit;		sv40-miR-S1-5p	[106]
Studies on EVs and whole viruses					
*JCPyV*	SVG-A, CPE cell supernatant	Ultracentrifugation; NTA, Immunoelectron microscopy, WB	CD9, CD81, annexin V, flotillin-1, TSG101	Whole-virus particle	[107,108,109]
*BKPyV*	Vero supernatant RPTEC supernatant	Ultracentrifugation; Immunoelectron microscopy	CD9, CD63, CD81	Whole-virus particle	[110]
*JCPyV*	Plasma	Exosomes extraction kit; NTA and WB, Immunoelectron microscopy	CD63, CD81, annexin II	Viral DNA and Vp1	[111]

RPTEC, renal proximal tubule epithelial cells; SVG, human fetal glial cells; CPE, choroid plexus epithelial cells; CSF, cerebrospinal fluid; NTA, nanoparticle tracking analysis; WB, Western blotting.
